# Laparoscopic diagnosis and extraction of an ingested fish bone that penetrated the stomach: A case report

**DOI:** 10.1097/MD.0000000000018373

**Published:** 2019-12-16

**Authors:** Zhi Zhang, Gang Wang, Zhigang Gu, Jie Qiu, Chuanfu Wu, Jianzhong Wu, Weixian Huang, Genhai Shen, Zhenghai Qian

**Affiliations:** a Department of General Surgery, Suzhou Ninth People's Hospital, Suzhou, China.

**Keywords:** fish bone, foreign body, gastrointestinal perforation, laparoscopic surgery

## Abstract

**Rationale:**

Foreign body ingestion is a common clinical event, but serious complication such as perforation is uncommon. Here we present a case of gastrointestinal perforation caused by fish bone, which was treated effectively and successfully by totally laparoscopic management.

**Patient concerns:**

A 63-year-old man who was admitted to our hospital with epigastric pain for 1 month. Computed tomography of the abdomen at the local hospital revealed a linear, hyperdense, foreign body in the lesser curvature of gastric antrum that had penetrated through the posterior wall of the gastric antrum.

**Diagnosis:**

The laparoscopic exploration found that a 2.5 cm × 0.3 cm fish bone had penetrated through the posterior wall of the gastric antrum.

**Interventions:**

A totally laparoscopic surgery was performed to remove the foreign body and repair the perforation eventually.

**Outcomes:**

After surgery, the patient underwent uneventful recovery and was discharged on postoperative day 7. During the 3 months of follow-up visit, the patient appeared healthy and did not report abdominal symptoms.

**Lessons:**

In this case, the advantages of laparoscopic techniques in the diagnosis and treatment of gastrointestinal perforation caused by foreign body was confirmed, and which may be considered as the primary choice in similar cases.

## 1 Introduction

Although inadvertent dietary ingestion of a foreign body is a common situation in our life, the majority of ingested foreign bodies pass through the gastrointestinal tract without symptoms spontaneously within one week, and only approximately 1% will occur gastrointestinal perforation, which is a rare clinical condition.^[1,2]^ According to the literature, fish bone is not only the most common foreign body in digestive tract, but also the most common cause of gastrointestinal perforation. And for most cases, gastrointestinal endoscopy and CT are recommended first to diagnose these unusual situations.^[3,4]^ Treatment for ingested foreign bodies usually includes endoscopic therapy, laparotomy and laparoscopy.^[5–7]^ We present a case of gastrointestinal perforation caused by fish bone, which was treated effectively and successfully by totally laparoscopic management.

## 2 Case presentation

**Figure 1:**
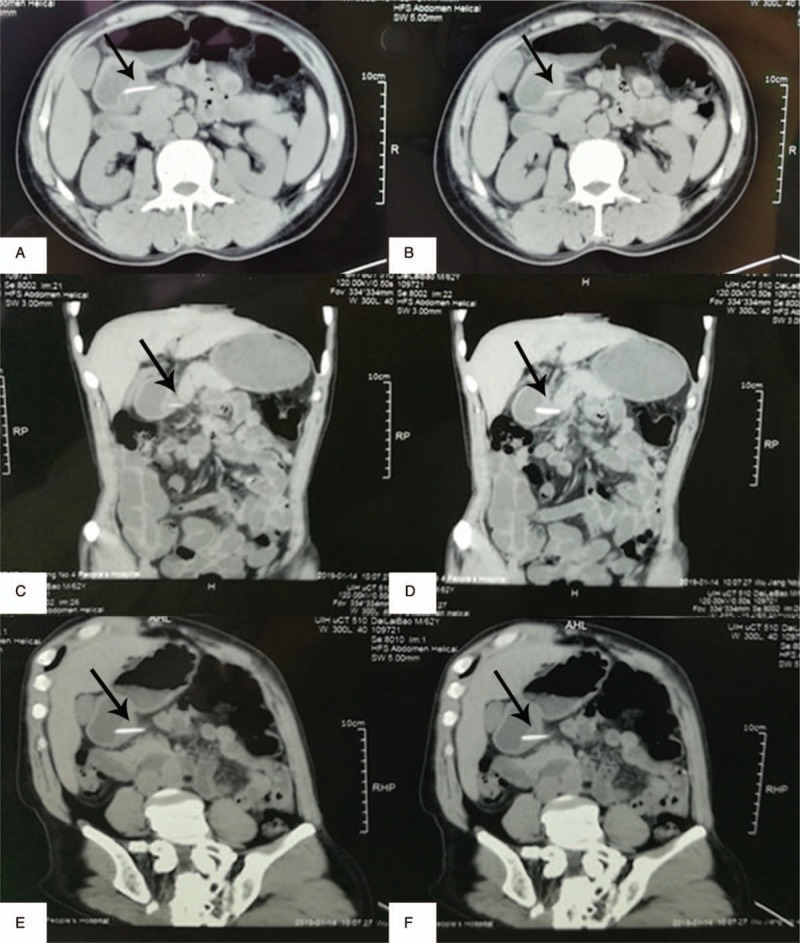
Abdominal CT showing a linear, hyperdense, foreign body (arrow) perforating the lesser gastric curvature. (A, B: Transverse section; C, D: Coronal section; E, F: Sagittal section).

**Figure 2:**
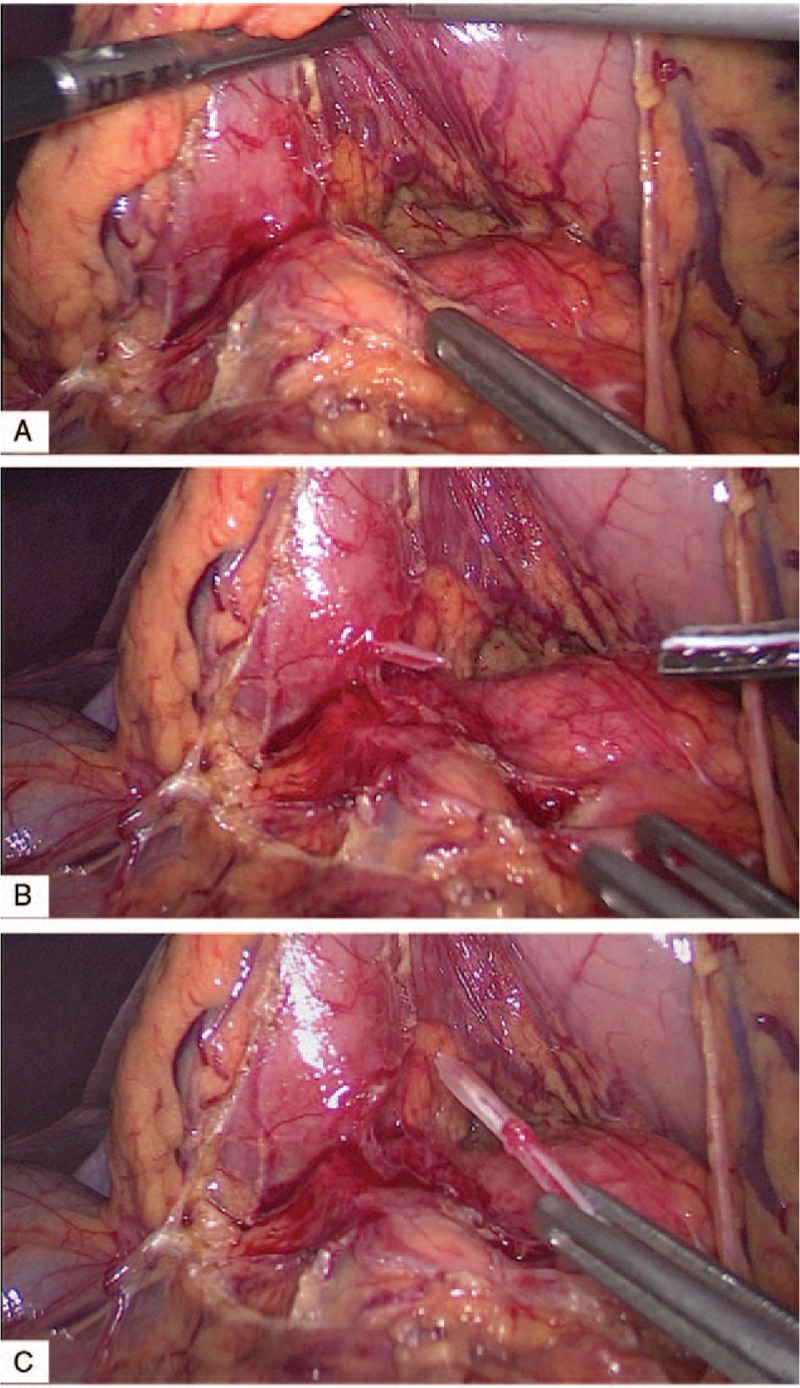
Laparoscopic extraction of an ingested fish bone. Severe adhesions were between the gastric antrum and the greater omentum (A). Foreign body perforating the lesser gastric curvature (B). Extraction of the foreign body from gastric antrum (C).

**Figure 3:**
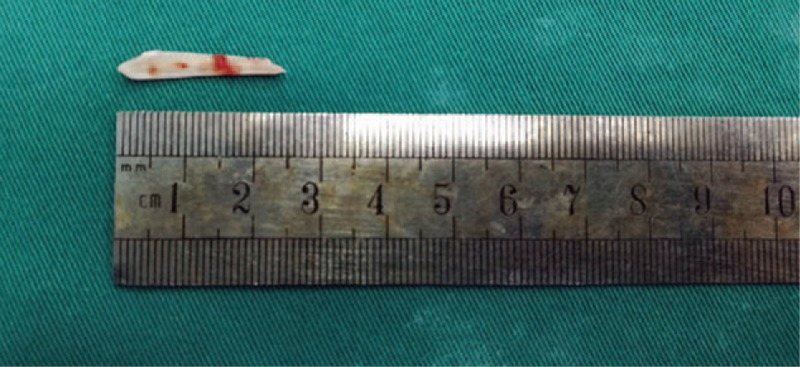
Operative view of the removed fish bone (approximately 25 mm long).

A 63-year-old man was admitted to our hospital with discomfort and upper abdominal pain that had started 1 month before. He had a history of hypertension and was treated with Levamlodipine Besylate Tablets. He was admitted in local hospital with 1-month history of intermittent pain that progressively worsened and was located in the epigastrium. The patient did not experience nausea, vomiting and diarrhea. Gastroscopy performed at another hospital revealed superficial gastritis with erosion. Computed tomography (CT) of the abdomen at the local hospital showed a linear, hyperdense, foreign body in the lesser curvature of gastric antrum that had penetrated through the posterior wall of the gastric antrum (Fig. 1A–F). He was then referred to our hospital for further treatment. On admission, the vital signs were stable and body temperature was 36.7°C. Physical examination revealed tenderness in the right upper quadrant and epigastrium. Laboratory investigations reveled a normal white blood cell count and other laboratory tests were found in normal reference ranges. We suspected that if the patient has a history of fish bone ingestion, As it is been a long time since the patient felt discomfort in the upper abdomen, the patient did not remember if there was a history of foreign body(such as fish bone, chicken bone or toothpick) ingestion. And because no foreign body was found in the gastroduodenum by gastroscopy, Hence, an explorative laparoscopic surgery was performed. The laparoscopic exploration found that severe adhesions were between the gastric antrum and the greater omentum, and we use an ultrasonic scalpel to dissect the adhesions carefully. Then, the foreign body was noted and safely removed laparoscopically (Fig. 2A–C), which was identified as a fish bone (length, 2.5 cm; diameter, 0.3 cm) (Fig. 3). At last, we repaired the perforation and cover the site of the penetrated gastric wall with the omentum. After surgery, the patient underwent uneventful recovery and was discharged on postoperative day 7. During the 3 months of follow-up visit, the patient appeared healthy and did not report abdominal symptoms.

## 3 Discussion

Inadvertent dietary ingestion of a foreign body is a common situation in our life, and most of cases occur in children, elderly persons or individuals with psychiatric problems, cognitive disorders, swallowing problems and alcohol ingestion.^[1,8–10]^ The most common types of gastrointestinal foreign bodies depending on different age group probably. Fish bones, chicken bones, toothpicks are common in adults, while nuts, fruit shells, coins and toys are common in children.^[11–14]^ However, the majority of ingested foreign bodies pass through the gastrointestinal tract without symptoms spontaneously within 1 week, and only approximately 1% will occur gastrointestinal perforation, which is a rare clinical condition.^[1,2]^ Most of foreign bodies that are causes of gastrointestinal perforation are fish bones reported in medical literature, and the stomach and the duodenum appear to be the most common regions of perforation of the gastrointestinal tract,^[15,16]^ although it tends to occur in sites of all segments of digestive tract.

According to the literature, only a few people who swallow foreign bodies can perceive or recall eating foreign bodies by mistake. It is very difficult to collect medical history. It has been reported that patients rarely recall any recent history of foreign body ingestion generally, as described in our case, making an early diagnosis clearly more difficult.^[3,10,17]^ Moreover, The clinical features of digestive tract perforation are different, some of them are acute abdomen, but most of them are non-specific chronic inflammation, abdominal mass or abscess, which is one of the causes of misdiagnosis and missed diagnosis.^[2,18,19]^ In our reported cases, the sharp end of the fish bone swallowed by the patient pierced the gastric wall under the action of gastric peristalsis. Because the gastric wall is thicker, it is likely that the perforation site will be gradually impacted and covered by the adjacent fibrin, omentum and surrounding tissues, which makes the clinical pictures more concealed.^[2,20]^

In general, abdominal X-ray plain film, B-ultrasound, CT and Gastrointestinal endoscopy can be used to diagnose foreign bodies in the digestive tract. Gastrointestinal endoscopy or CT scan are the preferred diagnostic tests to diagnose these unusual situations.^[2,21]^ Although gastrointestinal endoscopy is an indispensable examination, sometimes it was not useful for the event that had occurred considerably a long time ago, As in our case, no foreign body was found in the gastroduodenum by gastroscopy, as the fish bone had pierced the gastric wall. Hence, it makes the CT scan more important, which has high sensitivity and accuracy. Usually, CT will reveal a linear, hyperdense, foreign body.^[12,22]^ Moreover, CT is helpful for further confirming the location of foreign bodies and planning the surgical management.^[3,23]^

As for treatment of foreign bodies in the gastrointestinal tract, it is clear that early diagnosis and extraction are crucial to prevent bad complications. It has been reported that the treatment for ingested foreign bodies usually includes endoscopic therapy, laparotomy and laparoscopy.^[2,5,7]^ In our experience, when the foreign body has not completely penetrated the gastrointestinal wall, Endoscopic removal may be attempted firstly because of the advantages of relatively non-invasive operation. Otherwise, When a foreign body has pierced the gastrointestinal wall or caused peritonitis and abdominal abscess, laparoscopic or open surgery was recommended^.^^[24–26]^ And laparoscopic surgery has the advantages of relatively minor trauma, less pain, quick recovery and short duration of hospitalization when compared with laparotomy.^[21,27,28]^ But if the abdominal inflammatory adhesion is serious or laparoscopic surgery is unsuccessful, laparotomy should be considered.^[6]^ As in our case, because no foreign body was found in the gastrointestinal tract by gastroscopy, the fish bone was detected and removed by laparoscopic surgery eventually.

In summary, the present case and currently available literature demonstrate the significance of the diagnosis of gastrointestinal foreign bodies. Based on our experience, for most cases, gastrointestinal endoscopy and CT are recommended firstly, which not only offer location of foreign bodies but also show the relationship with the adjacent organs. When the diagnosis of digestive tract perforation caused by foreign bodies is clear, endoscopic therapy, laparoscopy, even laparotomy should be performed as soon as possible according to different situation. Moreover, in this case, the advantages of laparoscopic techniques were confirmed, and which may be considered as the primary diagnosis and treatment of choice in similar cases.

## Acknowledgments

We thank Songbing He for the proofreading and the correction of this manuscript.

## Author contributions

**Conceptualization:** Genhai Shen, Zhenghai Qian.

**Data curation:** Gang Wang.

**Formal analysis:** Zhigang Gu.

**Investigation:** Jie Qiu.

**Methodology:** Chuanfu Wu.

**Validation:** Jianzhong Wu, Weixian Huang, Zhenghai Qian.

**Writing – original draft:** Zhi Zhang.

**Writing – review & editing:** Zhi Zhang.

Zhenghai Qian orcid: 0000-0001-5125-4164.
